# Multi-feature machine learning model for automatic segmentation of green fractional vegetation cover for high-throughput field phenotyping

**DOI:** 10.1186/s13007-017-0253-8

**Published:** 2017-11-21

**Authors:** Pouria Sadeghi-Tehran, Nicolas Virlet, Kasra Sabermanesh, Malcolm J. Hawkesford

**Affiliations:** Plant Science Department, Rothamsted Research, Harpenden, AL5 2JQ UK

**Keywords:** Field phenotyping, Learning-based segmentation, Fractional cover, Field Scanalyzer, RGB images

## Abstract

**Background:**

Accurately segmenting vegetation from the background within digital images is both a fundamental and a challenging task in phenotyping. The performance of traditional methods is satisfactory in homogeneous environments, however, performance decreases when applied to images acquired in dynamic field environments.

**Results:**

In this paper, a multi-feature learning method is proposed to quantify vegetation growth in outdoor field conditions. The introduced technique is compared with the state-of the-art and other learning methods on digital images. All methods are compared and evaluated with different environmental conditions and the following criteria: (1) comparison with ground-truth images, (2) variation along a day with changes in ambient illumination, (3) comparison with manual measurements and (4) an estimation of performance along the full life cycle of a wheat canopy.

**Conclusion:**

The method described is capable of coping with the environmental challenges faced in field conditions, with high levels of adaptiveness and without the need for adjusting a threshold for each digital image. The proposed method is also an ideal candidate to process a time series of phenotypic information throughout the crop growth acquired in the field. Moreover, the introduced method has an advantage that it is not limited to growth measurements only but can be applied on other applications such as identifying weeds, diseases, stress, etc.

**Electronic supplementary material:**

The online version of this article (10.1186/s13007-017-0253-8) contains supplementary material, which is available to authorized users.

## Background

An average 2.4% annual yield increase needs to be achieved in order to meet the required estimated doubling of crop production by the year 2050. However, the average rate of increase for the four global key crops ranges from 0.9 to 1.6% [28]. To bridge this gap, the breeding process must be accelerated. High-throughput field phenotyping enables the capacity for rapid and large scale evaluation of crop performance in agriculturally relevant environments which will help to accelerate the breeding process and ultimately, the rate of genetic improvement [1].

Digital RGB cameras are the most common tool used for field phenotyping due to their high-resolution, low cost and portable size. RGB cameras constitute a simple tool that provides a non-destructive, non-invasive and generally a high-throughput approach to collect information about canopy development and health status. RGB images have been used to provide plant morphological information using stereoscopic approaches [17], and to estimate net primary production using intensity of the reflectance of each of the red, green and blue channel [11, 16]. In recent years, RGB images have been extensively used to estimate fractional vegetation cover to study plant responses to water stress [7, 35], nitrogen nutrition [19, 20, 32] or disease [38], as well as for the detection of weeds [24], for plant biomass estimation [6, 21] and for yield [9].

Fraction vegetation cover (FVC) is derived from images collected from the nadir position [8, 10]. FVC is assessed as the ratio of green vegetation pixel to the total number of pixels for a given area. Effective segmentation algorithms to extract green vegetation pixels have been implemented in numerous studies over the last decades, from using simple colour indices to machine learning approaches [2, 12, 13, 24].

Colour is the most popular feature for visual-based plant segmentation due to low computational cost, particle occlusion, robustness, and resolution changes. Colour properties are easily extracted and relatively constant under viewpoint changes. However, colour-based techniques have a problem in maintaining colour constancy between and within images of the same object, simply due to changes in illumination conditions, inter-reflections with other objects, shadows, etc. These effects may be minimised by using colour space transformation, such as YCbCr, HSL, HSV, CIELab and CIELuv [6] or using red, green and blue band combinations to increase the contrast between background (soil) and foreground (vegetation). Indices such as excess green (ExG) or excess green minus excess red (ExGR) were used in conjunction with automatic and positive thresholds, respectively, for an automatic background/foreground segmentation [24]. Alternatively, other colour indices have been developed to improve the quality of the segmentation and to handle ambient illumination [13].

As classical methods with fixed thresholds (determined manually for each image by an operator) have difficulties in segmenting vegetation from background efficiently, automated thresholding methods have been developed based on bimodal distribution of green pixel intensity (or the corresponding channel when a colour space is used). The bimodal distribution corresponds to the background and foreground pixels. A Gaussian mixture model (GMM) is used to separate the pixel distribution of the foreground pixels from the background and automatically define a threshold [8, 22, 29]. However, when vegetation is sparse or the canopy is nearly fully closed, the bimodal distribution is not apparent [8]. Thus, GMM is likely to fail to discriminate vegetation from background, especially when weeds or algae are present in the captured image.

Several machine learning methods have been proposed to address such limitations. For example, [37] used mean-shift clustering to segment the green vegetation of a crop canopy. However, each image sample was manually pre-segmented into separate regions; thus, it is not a practical solution for automated high-throughput phenotyping applications where hundreds of images are captured each day with high temporal resolution. [25] used ExR and ExG as inputs for fuzzy clustering to classify plants, soil and crop residue regions. These approaches achieved only 69% accuracy to classify plants in bare soil and failed for plants in corn and wheat residues. [30] proposed an adaptive segmentation algorithm for outdoor image segmentation. Although their method showed some level of adaptiveness with illumination changes, it generated noise in direct sunlight and failed to segment the majority of vegetation. [12] utilised a decision tree classification method to address the specular reflection on plant leaves. Their method segmented a high level of vegetation from their digital image of a single plant growing outdoor when compared to classical methods (such as ExG or ExGR), as well as in various ambient illumination conditions. However, in order to be suitable for phenotyping field-grown crops, any developed method for image segmentation must be extensively tested at canopy-scale throughout the entire crop life cycle. [3] developed a supervised approach with morphological modelling. The method is developed further in [2] by adding clustering approach to the process. Although the results outperformed colour index-based techniques such as ExG and ExGR and learning models such as GMM, the proposed method requires too many steps to execute and is prone to error and not suitable for daily operation for high-throughput phenotyping.

### Challenges

To reliably classify vegetation from background (e.g. soil, rock) within an image, several challenges must be overcome when implementing a modelling algorithm. A key challenge when acquiring a time series of digital images in the field is the wide range of colour temperatures of ambient light. Changes in colour temperature depend on the sun position during the day and amount of cloud cover. [33] showed that colour temperature changes from 3400 (sunset) to 9500 K (north sky light) in daylight. Furthermore, the colour temperature at midday can fluctuate between 6000 and 9300 K as result of sunny or overcast skies. Such variation can result in poor performance of colour threshold-based and colour index-based approaches in segmenting vegetation from images captured in the field. The other issues are shadows and/or distribution of illumination variations. Illumination levels may vary between 102,000 lux (maximum sunlight) to 10,000 lux (shadows in sunny day) [33]. In general, modern cameras with automatic exposure times and ISO values can handle the illumination distribution in an image to some extent; however, too much variation in the camera parameters (e.g. exposure) leads to false colour identification of objects. Apart from extremely dark or light conditions, humans are able to recognise non-uniformly illuminated scenes, due to their capability of thresholding each individual part locally. However, most of the aforementioned image processing methods failed to cope with this variation.

Colour transformation provides an ideal solution to minimise the shadow effect. Colours in an image are split into brightness and chromaticity. Then, an assumption is made that the chromaticity remains almost constant when the brightness changes if a pixel is part of a shadow. Moreover, in some scenarios, for example, after a rainy day, the sun on the wet surface (e.g. leaf) may contribute to increase reflection and cause difficulty in segmenting plants precisely. The main reason is that reflections retain colour, texture, and edge information that are missing in shadows. Thus, most algorithms that rely on colour or texture will most likely fail to distinguish the plant’s reflected surface from the background.

Similarly, detection of specular reflective regions is hardly achievable using classical segmentation based on colour properties, as those regions display a saturated signal (white spot) in all the RGB channels. Ideally, data must be captured in optimal light conditions. However, as illumination conditions under temperate climates may be uncertain over a day and will inevitably change through the season, segmentation algorithms must be robust enough to cope with dynamic illuminations. In recent years, intense efforts have been driven by the scientific community of crop and computer scientists to develop new techniques to process field phenotyping data. Machine learning is a promising multidisciplinary approach to data processing as it combines statistics, optimisation and modelling techniques.

This paper describes a machine learning technique to analyse field data (digital images) for the new generation of phenotyping platforms. The described supervised method is capable of learning from environmental conditions with a high level of adaptiveness and is suitable for high-throughput analysis in terms of processing time.

## Methods

**Fig. 1:**
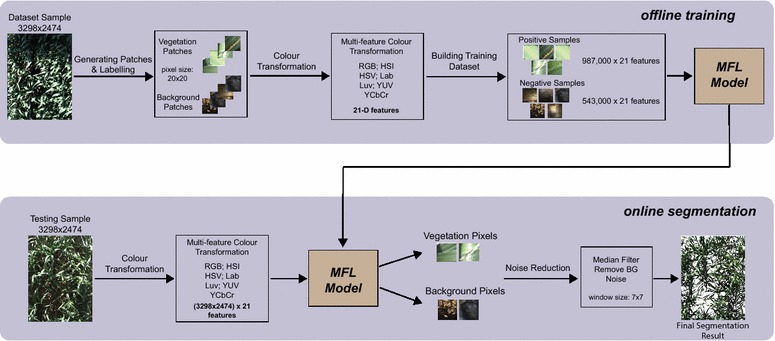
Schematic representation of the method

The entire process of the proposed method includes the following steps (Fig. 1):

- Acquisition of digital images in time series
- Extraction of multi-feature colour transformation
- A supervised classification model to label pixels as foreground or background
- Noise reduction using median filtering

**Fig. 2:**
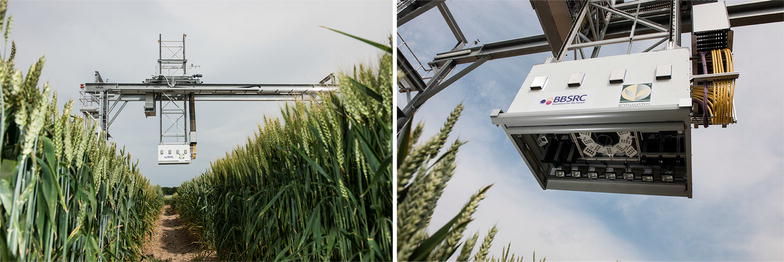
Field Scanalyzer. (Left) The Field Scanalyzer phenotyping platform at Rothamsted Research showing (Right) the cameras within the camera bay directed down, perpendicular to the ground

### Field experiment and image acquisition

Six wheat cultivars (*Triticum aestivum* L. cv. Avalon, Cadenza, Crusoe, Gatsby, Soissons and Maris Widgeon) were grown in the field at Rothamsted Research, Harpenden, UK. All cultivars were sown at a planting density of 350 seeds/$\hbox {m}^{2}$ on 20 October 2015 (Autumn) and harvested on 27 August 2016 (Summer). Nitrogen (N) treatments were applied as ammonium nitrate in the spring, at rates of 0 kg $\hbox {ha}^{-1}$ (residual soil N; N1) 100 kg $\hbox {ha}^{-1}$ (N2) and 200 kg $\hbox {ha}^{-1}$ (N3).

The digital camera (colour 12 bit Prosilica GT3300) on the Field Scanalyzer phenotyping platform (LemnaTec GmbH; Virlet et al. [31]) was used to acquire all digital images (Fig. 2). The camera was perpendicular to the ground and positioned to maintain a 2.5 m distance between the camera and canopy, representing an area of $\approx 0.5\,{\hbox {m}^2}$. The digital camera was set up in auto-exposure mode to partly compensate for outdoor light changes. Wheat canopies were imaged 1–2 times per week. From the digital image library, 33 time points between sowing and harvest were randomly selected to represent the entire crop lifecycle. Additionally, on 2nd April 2016 [165 days after sowing (DAS)] a single plot were imaged at four time points throughout the day (10:16 AM, 12:44 PM, 3:36 PM, 5:03 PM).

**Fig. 3:**
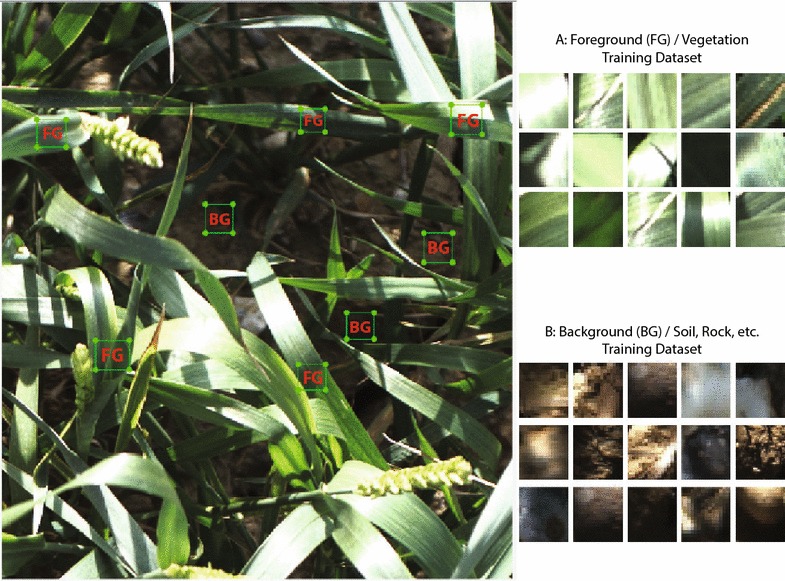
Training patches. Examples images from foreground (FG) and background (BG) training patches in various illumination conditions

**Fig. 4:**
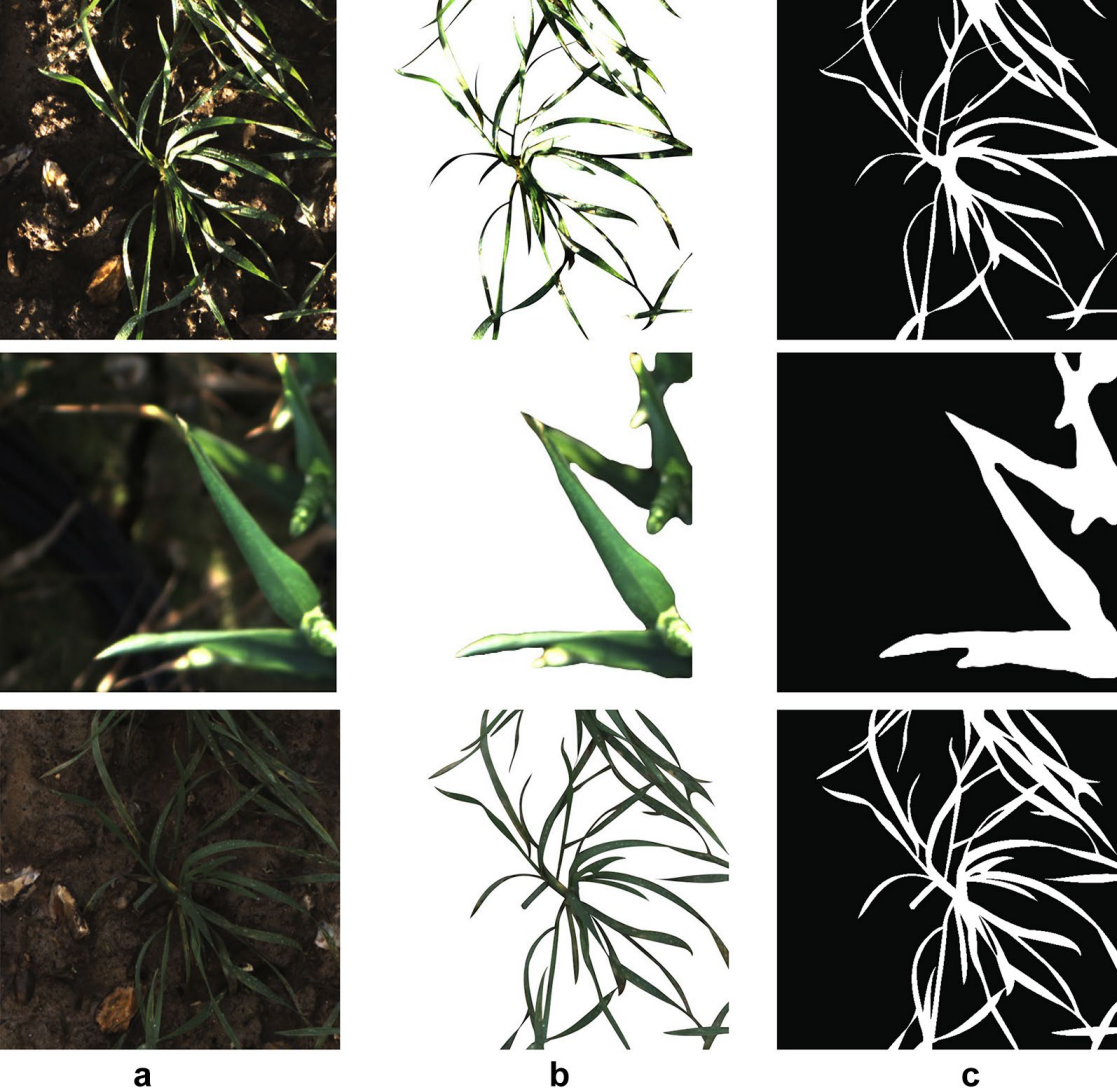
Examples of test images and their corresponding ground truths. The test images randomly selected from the image dataset in different illumination conditions. **a** original image, **b** reference image segmented manually, **c** binary image of the reference image

On the 21st June 2016 (245 DAS), leaf area index was measured with a ceptometer, (LAI-2200C Plant Canopy Analyser $Licor^{\textregistered }$) on each of the 54 plots of the experiment. Three measurements were taken at each plot with a $90^\circ$ view-restricting cap, one above the vegetation and two below. The above vegetation reading was performed to correct the below vegetation reading for light intensity. The correction is automatically performed by the ceptometer and the Leaf Area Index (LAI) was extracted using FV2200 software (Licor Bioscience, v2.1.1). For each plot, two LAI values were averaged before comparing with canopy closure data obtained from RGB images collected the same day.

### Multi-feature supervised machine learning approach

Low-level cognitive functions are important in computational intelligence, which also involves discovery of structures in data analysis, object recognition and segmentation. The techniques introduced to address the problem can be divided into two main groups of supervised and unsupervised learning. They include Bayesian networks, statistical and kernel methods as well as evolutionary, fuzzy and neural approaches. In supervised techniques, the information is supplied by pre-defined class labels and pre-trained samples. Conversely, the unsupervised pattern representations do not require any pre-trained samples. A supervised multi-feature model is developed which is capable of training a model in different field conditions and labelling each image pixel as background or vegetation regardless of environmental conditions in the field.

### Feature extraction

Visual features are fundamental in processing digital images to represent an image content. A set of good features should contain sufficient discrimination power to discriminate image contents. In this paper, colour properties are used as the main features to segment plants and monitor canopy coverage. Colour properties are extracted directly from pixel densities over the whole image and carry enough information about an image to discriminate plants from the background. In addition, colour features are sufficiently robust to handle background complications and invariants to the size, orientation and partial occlusion of the canopy image. In any colour-based method, having a colour consistency is an important factor. To maintain this consistency in a colour space, illumination conditions should not be changed; however, this is not achievable in the field with illumination changes, shading and a cluttered background (e.g. soil). In order to achieve an efficient system, a multi-dimensional feature is used to describe different properties of an image which also gives the ability to resist noise induced variations. Six colour spaces known as L*u*v*, L*a*b*, HSV, HSI, YCbCr, and YUV are used to extract the colour properties of each pixel; thus, the final size of the feature vector is equal to 21 elements:

1 $$\begin{aligned} F&= \left\{ f_1^R,f_2^G,f_3^B,f_4^{{L^*}},f_5^{{a^*}},f_6^{{b^*}},f_7^{{L^*}},f_8^{{u^*}},f_9^{{v^*}},f_{10}^H,f_{11}^S,f_{12}^V,f_{13}^H,f_{14}^S,\right. \nonumber \\&\quad \left. f_{15}^I,f_{16}^y,f_{17}^{{C_b}},f_{18}^{{C_r}},f_{19}^Y,f_{20}^U,f_{21}^V \right\} \end{aligned}$$

### Training and testing the model

The fundamental part in any decision making supervised classification system, such as the one proposed in this paper, is how to specify the output or *action*, determined based on a given sets of inputs or training data. Therefore, good selection of training datasets will lead to better performance in classification model. A wide range training dataset is selected to cover different field conditions. The classification system is formulated as a two-class model. The first class (positive training samples) contains image patches manually labelled from different wheat varieties at various growth stage and lightning conditions, such as sunny, cloudy, and rainy conditions (Fig. 3a); whereas, the second class (negative training samples) contains background images manually segmented from soil, rocks, etc. (Fig. 3b). The positive training dataset contains 2429 Foreground (FG) patches ($N_p = 2429$), while the negative training dataset contains 1332 patches ($N_n = 1332$). Each training patch is resized to $20\times 20$ pixel size then transformed into multiple colour spaces. Each image patch is represented by a $x = N \times n$ matrix; where *N* is number of pixels in each patch ($20\times 20$) and *n* is colour features (*n* = 21). Therefore, the training dataset includes $987,000 \times 21$ positive samples and $543,600 \times 21$ negative samples.

**Fig. 5:**
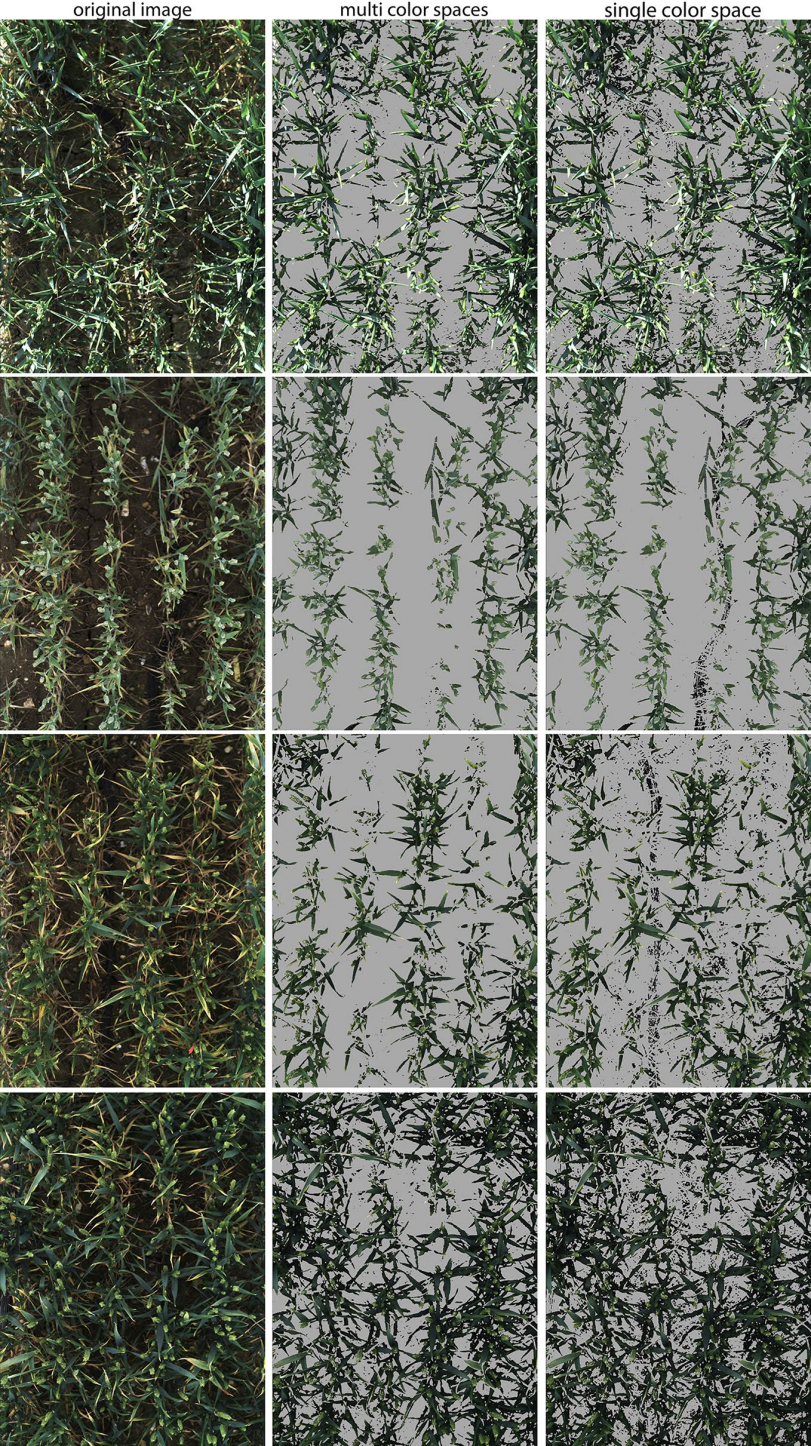
Multi-feature versus single feature. Comparison of segmented images of supervised learning model with single colour space versus multiple colour spaces

**Fig. 6:**
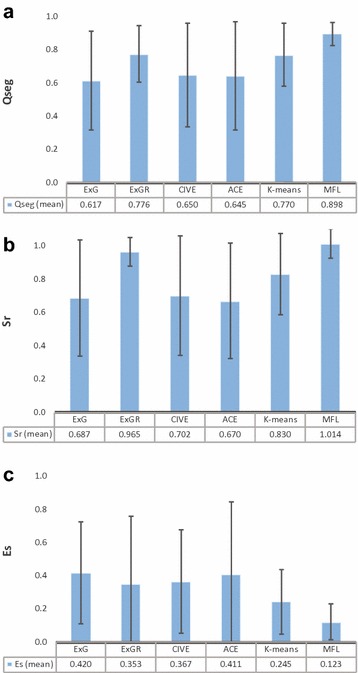
Comparison of the mean accuracy rate ($Q_{{ seg}}$, $S_{r}$, and $E_{s}$). Comparison of different approaches by segmentation quality for ExG, ExGR, CIVE, ACE, *K*-means, and the proposed method, MFL. The bar indicates the standard deviations

The random forest classifier [14] is then used to train the model based on the generated matrix obtained from the positive and negative training dataset. Random forest is an intuitive decision rules classifier which can handle non-linear features and take into account variable interactions. In our application, two-classes are trained by labelling the foreground/vegetation patches as (+ 1), and the background as (0). The random forest classifier used multiple decision trees conducted into one meta classifier and was then used to make predictions. Each decision tree is trained individually and their probability distributions are aggregated to receive the final classification to label each pixel as vegetation (+ 1) or background class (0), which generates a binary image.

### Noise reduction

A median filter is applied to minimise the noise and remove the result of misclassification over the binary image [15]. In this work, a window size of seven pixels slides over the entire image, pixel by pixel. Then, the pixel values from the window are sorted numerically and replaced with a median value of neighbouring pixels.

## Experimental results

The performance of the developed machine learning method was evaluated through several experiments under changing light conditions in a day, by comparing to manual measurement as well as throughout the canopy lifecycle. The algorithms were developed in MATLAB (Mathworks Inc.) as well as python using OpenCV [5] and scikit-learn [27] python packages. The process of segmenting an image with an original resolution of $3298 \times 2474$ only takes 3.4 s on a Windows 10 PC with 6-core Intel Xeon processor (3.60 GHz) with 32 GB RAM. Although the outcomes from MATLAB and python are almost identical, all the results and comparisons presented in this paper are under python development.

The introduced techniques were also compared with the three well-known colour-index methods, ExG, ExGR [24], and CIVE [18], as well as two unsupervised learning methods known as ACE [8] and *K*-means clustering techniques. ExG (Excess Green Index) was originally proposed by [34] to provide a clear contrast between plant and soil: $ExG = 2 \times G - R - B$. [24] used an automatic thresholding method known as the Otsu method [26], which enabled background and foreground segmentation based on the bimodal distribution of the pixel. ExGR combines ExG and ExR (Excess Red Index) to improve performance of ExG: $ExGR = ExG - \left( {1.4 \times R - G} \right)$ [23, 24]. The authors added a positive threshold to the index to remove residual background pixels and achieved a higher performance compared to the ExG method with an Otsu threshold. Colour Index of Vegetation Extraction (CIVE) was proposed by [18] to evaluate crop growing status by providing a greater emphasis on the green area: $CIVE = 0.441R - 0.811G + 0.385B + 18.78745$. Similar to ExG, the Otsu method is used for automatic segmentation of vegetation from a soil background. When ExG, CIVE and ExGR are subsequently mentioned in this paper, they will be referred to their respective thresholds, Otsu and positive threshold respectively. In addition to the three colour index-based approaches mentioned earlier, the performance of two learning based models are compared with the proposed approach. Automated canopy estimator (ACE) is used an unsupervised segmentation process to produce accurate estimate of fractional vegetation cover using GMM [8]. It should be noted that the ACE results presented in this paper are based on the free software provided by the authors at http://173.230.158.211. Another unsupervised learning model evaluated in this paper is *K*-means clustering developed to group pixels in digital images under a transformed L*u*v* colour space. A canopy is partitioned into *k* clusters ($k=20$). The − u* and + v* axis indicate where green colour falls; thus, green pixels distributed close to the negative value of u* and positive value of v*, contain vegetation. Therefore, the pixels which do not satisfy the following condition (Eq. 2) are considered as background.

2 $$\begin{aligned} \left\{ \begin{array}{l} {p_i} {\textit{ is background if else}}\\ {p_i} {\textit{ is foreground if }} c_i^{{u^*}} < 0; c_i^{{v^*}} > 0 \end{array} \right. \end{aligned}$$

where $p_{i}$ is an image pixel of *i* ^th^ cluster; $c_{i}$ is a cluster centre

### Evaluating the segmentation accuracy

The accuracy of all methods were evaluated with the reference images in which the vegetation was manually segmented using *Photoshop* (Adobe Systems Incorporated, San Jose, CA, USA). Due to the complexity of the tested images, special care was put into segmenting vegetation appropriately as a fully manual manipulation. Figure 4 shows three examples of test images and manually segmented reference images for ground truthing. Three quality factors known as $Q_{{ seg}}$, $S_{r}$ and an error factor $E_{s}$ [24, 36] are used to assess the segmentation accuracy using the following Eqs. 3, 4, and 5.

3 $$\begin{aligned} Q_{{ seg}}= & {} \frac{\sum _{i,j=0}^{i,j=h,w}(S(p)_{i,j}\cap R(p)_{i,j})}{\sum _{i,j=0}^{i,j=h,w}(S(p)_{i,j}\cup R(p)_{i,j})} \end{aligned}$$

4 $$\begin{aligned} S_{r}= & {} \frac{\sum _{i,j=0}^{i,j=h,w}(S(p)_{i,j}\cap R(p)_{i,j})}{\sum _{i,j=0}^{i,j=h,w}(R(p)_{i,j})} \end{aligned}$$

5 $$\begin{aligned} E_{s}= & {} \frac{\sum _{i,j=0}^{i,j=h,w}(S(p)_{i,j}\cap R(!p)_{i,j})}{\sum _{i,j=0}^{i,j=h,w}(R(p)_{i,j})} \end{aligned}$$

where *S* is the segmented plant ($\hbox {p}=255$) or background pixels ($\hbox {p}=0$). *R* is the reference image manually segmented. Indices *i*, *j* are the pixels coordinate, and *h*, *w* are the height and width of the image, respectively. The accuracy is based on logical operations, logical *and* ($\cap$), logical *or* ($\cup$) and logical *not* (!), compared on a pixel-by-pixel basis of the reference image *R* and segmented image *S*.

$Q_{{ seg}}$ is based on both plants and background regions within the range of values 0 and 1. It illustrates the consistency between the segmented image *S* and the reference image *R* on pixel-by-pixel basis where value 1 represents a perfect outcome. Similarly, $S_{r}$ measures the consistency within the image region of plant pixels and $E_{s}$ represents the portion of misclassified plant pixels relative to true total plant pixels.

### Single colour space versus multiple colour spaces as input(s) for the learning model

In this section, the performance of the learning model using single colour space with three features is compared to multiple colour spaces with 21 features as described in the feature extraction section. Table 1 illustrates the performance comparison between the two methods. It should be noted that L*a*b* colour space is used as a single input to meet the requirement of uniformity of distribution of colour [4]. It is device-independent and proved to perform well in segmenting vegetation under uncontrolled outdoor illumination conditions [3, 8].

**Table 1:** Comparison of the mean accuracy rate ($Q_{{ seg}}$, $S_{r}$, $E_{s}$) between multi-colour spaces and single colour space

	Multi colour spaces	Single colour space
*Q*_*seg*_	0.922 (0.019)	0.885 (0.087)
*S*_*r*_	1.029 (0.047)	1.025 (0.042)
*E*_*s*_	0.088 (0.022)	0.143 (0.131)

As shown in Table 1, using multiple colour spaces is more robust to background noise and outdoor illumination changes. While both approaches displayed similar $S_{r}$ values (1.029 +/− 0.047 and 1.025 +/− 0.042 in multi and single colour space, respectively), the multi-colour spaces had higher mean quality factor of 0.922 with lower standard deviation 0.019 as opposed to single colour space (0.885 +/− 0.087). In addition, multi-colour spaces had the lower rate of misclassified plant pixels (0.088 vs. 0.143) with lower standard deviation (0.022 vs. 0.131). As a result, multi-feature colour spaces (MFL) was selected as the optimum technique and compared with the state-of-the-art vegetation segmentation described in this work (Fig. 5).

**Table 2:** Monitoring of various image segmentation methods handling of ambient illumination changes within a single day

Time point	Par test	ExG	ExGR	CIVE	ACE	K-means	MFL
10:16 AM	999	0.105	0.328	0.100	0.333	0.351	0.279
12:44 PM	1352	0.128	0.313	0.126	0.311	0.363	0.258
3:36 PM	1004	0.146	0.322	0.170	0.457	0.330	0.258
5:03 PM	139	0.317	0.320	0.335	0.350	0.332	0.273
Avg		0.174	0.321	0.183	0.363	0.344	0.267
SD		0.097	0.006	0.105	0.065	0.016	0.011
CV(%)		56%	2%	58%	18%	5%	4%

### Comparison of image segmentation techniques with the ground truth segmentation

**Fig. 7:**
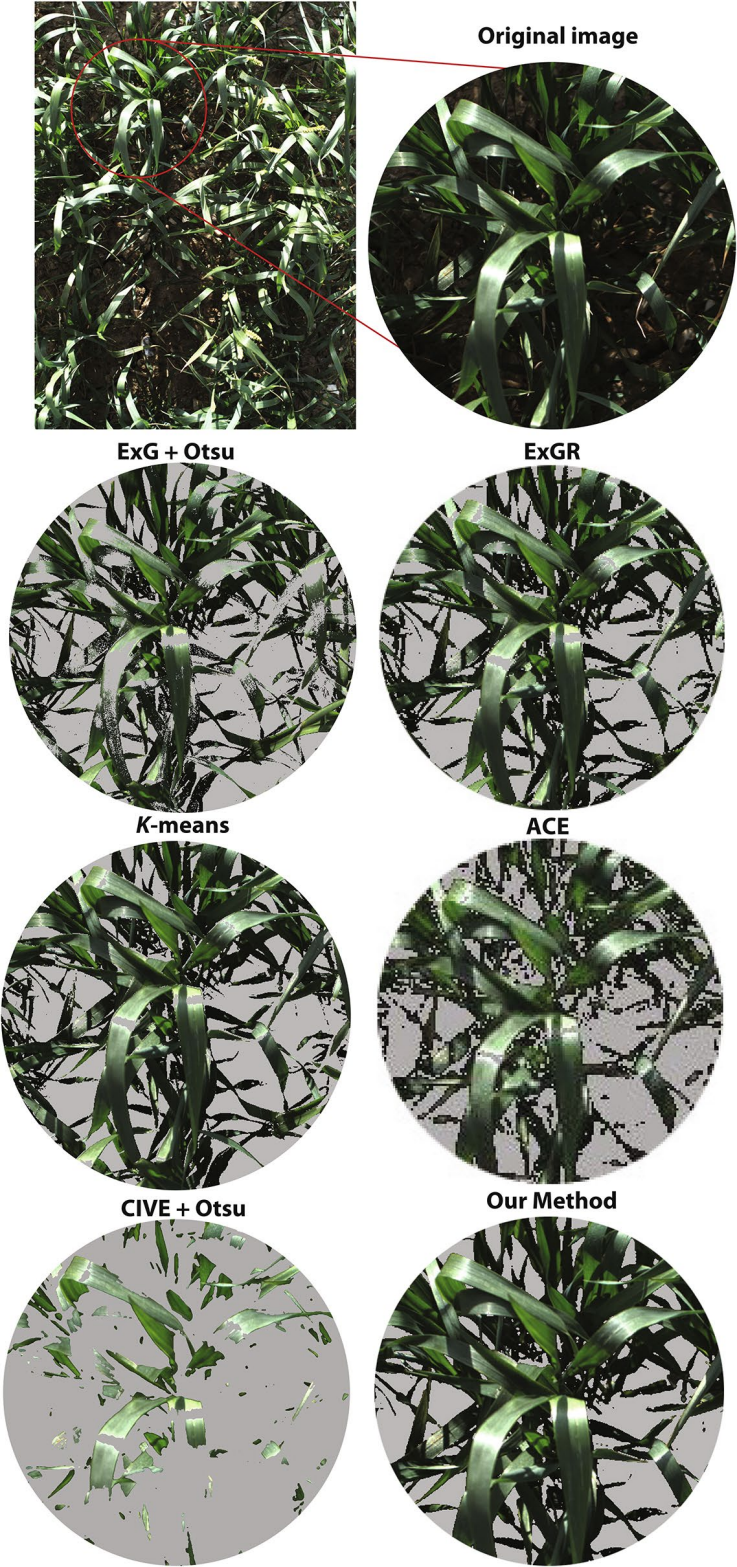
An example of vegetation segmentation by ExG, ExGR, CIVE, ACE, K-means, and our method

**Fig. 8:**
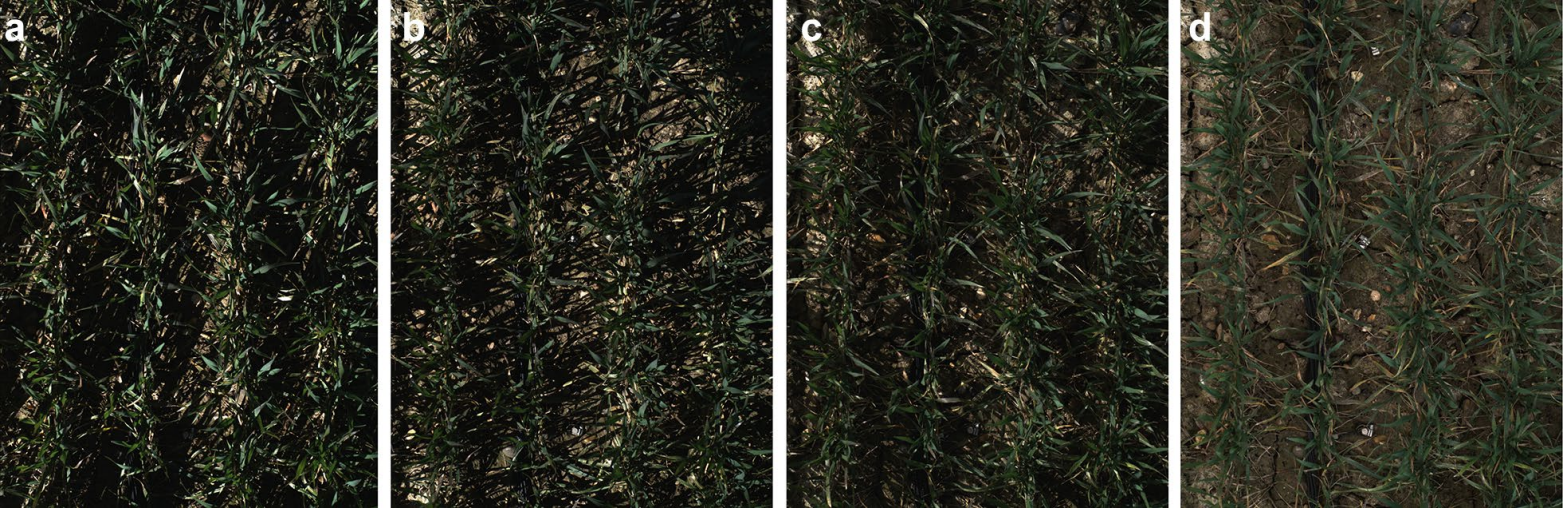
Digital images of a single section of a wheat plot (*Triticum aesvtivum* L. cv. Soissons) and the vegetation extracted using various image segmentation methods. Images were captured 165 DAS at **a** 10:16 AM, **b** 12:44 PM, **c** 3:36 PM, **d** 5:03 PM

Comparison of the accuracy rate of segmentation ($Q_{{ seg}}$, $S_{r}$, $E_{r}$) of the proposed method with five other methods is presented in Fig. 6. Ten images were randomly selected under varying conditions of spectral reflections and background noise on different days (Figs. 7, 8). As shown in Fig. 6a, ExG, CIVE and ACE had the lowest $Q_{{ seg}}$ values (0.617, 0.65 and 0.645, respectively) with the highest standard deviation (0.299, 0.314 and 0.327, respectively). ExGR and *K*-means came second and third with average values of 0.776 and 0.77 and standard deviation 0.169 and 0.19, respectively. Nevertheless, MFL had the highest $Q_{{ seg}}$ mean value of 0.898 and the lowest standard deviation, 0.07. MFL also had the highest $S_{r}$ mean value and lowest standard deviation along with ExGR with 1.014 and 0.965, respectively (Fig. 6b).

ExG and CIVE had the highest misclassified pixels ($E_{s}$) while *K*-means and ExGR came second and third with 0.245 and 0.353, respectively. MFL performed the best with the lowest misclassified segmented pixels values of 0.123 (Fig. 6c).

**Fig. 9:**
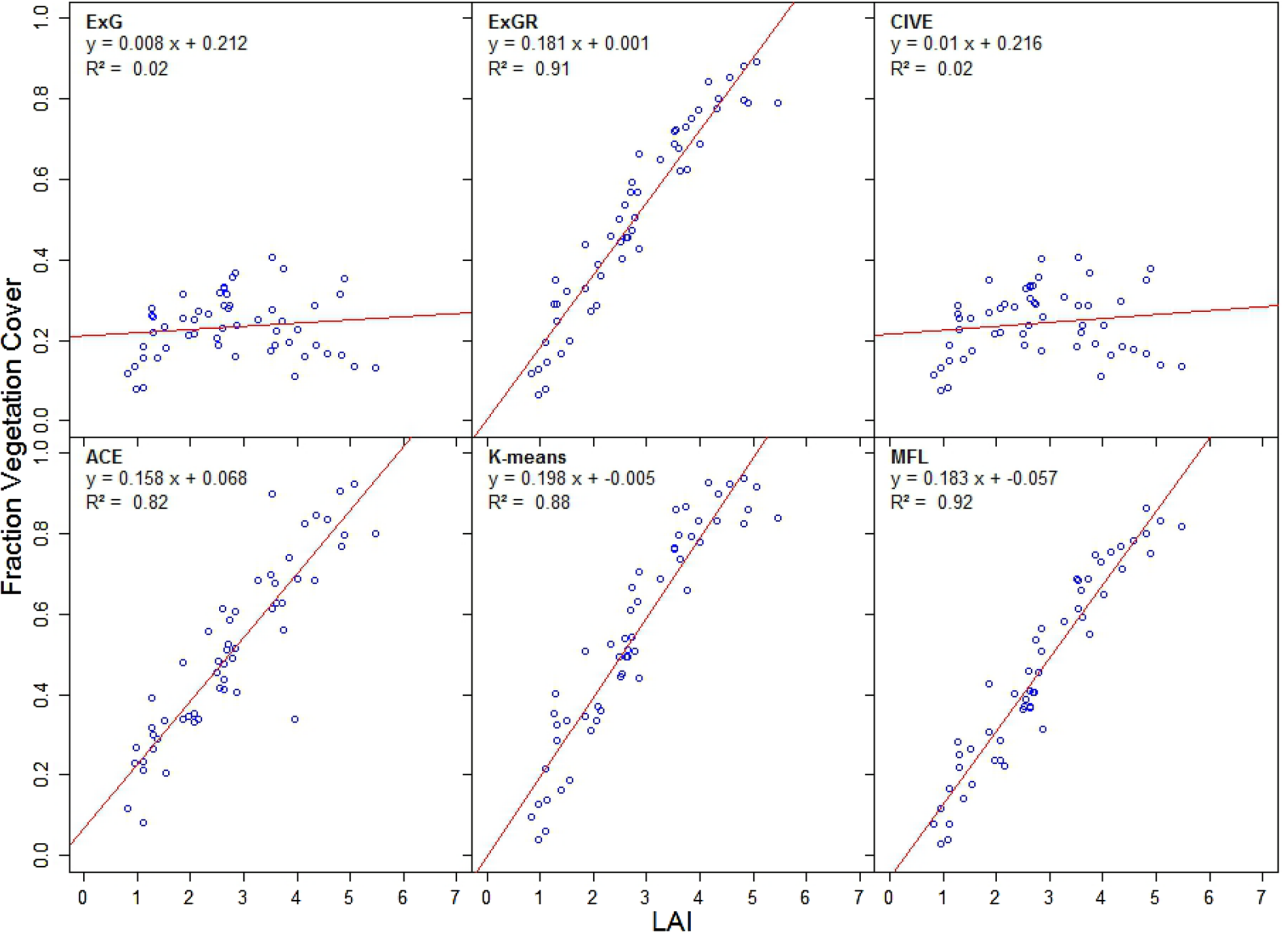
Comparison of manual canopy cover estimates of 54 wheat plots determined using leaf area index (LAI) with the automatic methods, ExG, ExGR, CIVE, ACE, K-means, and MFL

**Fig. 10:**
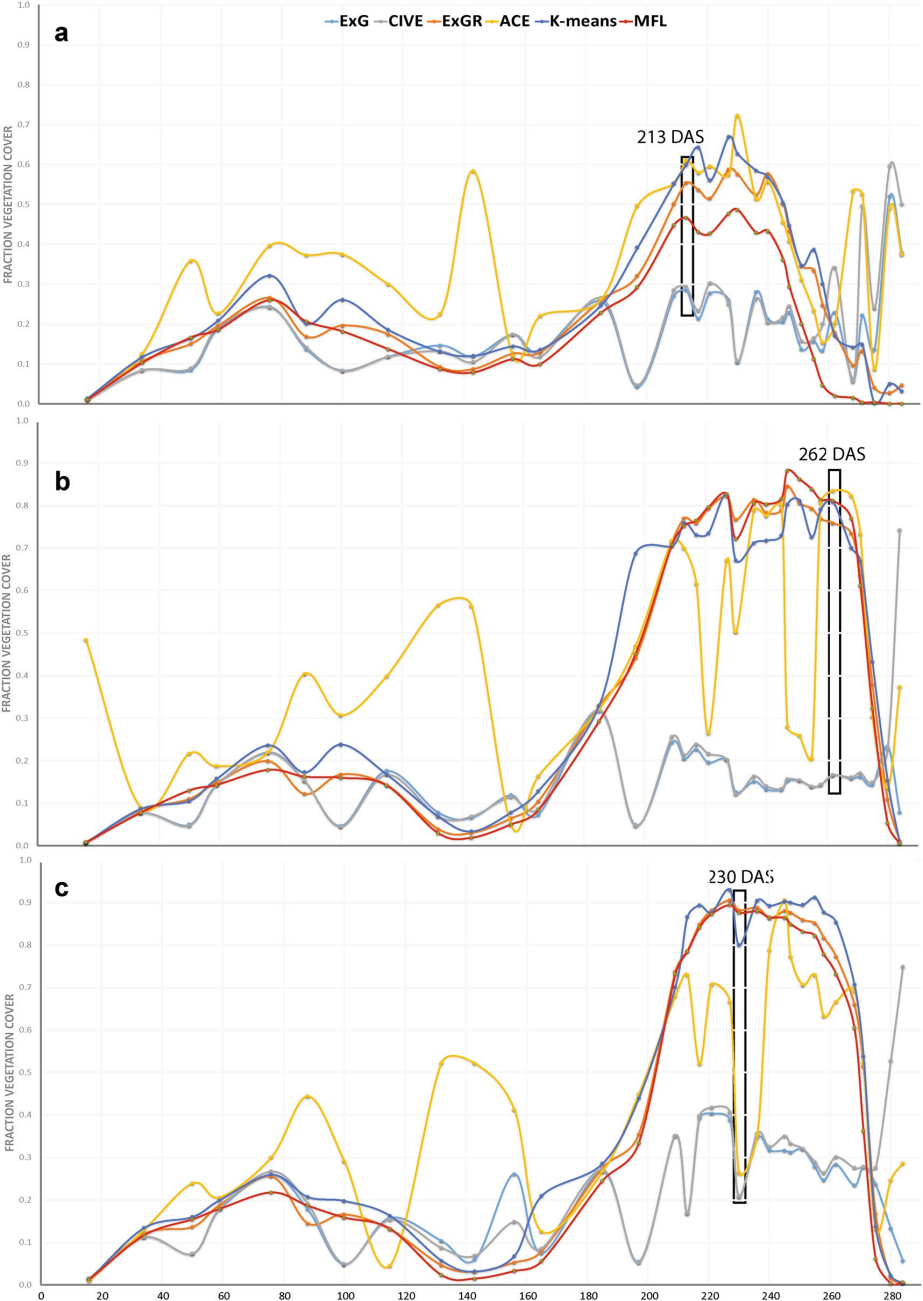
Canopy cover estimates. Comparison of the segmentation results for canopy cover at 33 random time points of the UK 2015–2016 growing season. **a** Crusoe, **b** Gatsby, **c** Widgeon

### Vegetation segmentation over illumination changes during a day

The performance of all methods in various ambient illuminations was assessed (Table 2). A screenshot of a Soissons plot was captured at different times of the day 165 DAS (April 2016). CIVE, ExG and ACE showed the highest coefficient of variations (CVs) with 58, 56 and 18%, respectively, while *K*-means, MFL and ExGR had the lowest coefficient of variation, below 5% over the day (5, 4 and 2% respectively).

### Automatic vegetation segmentation versus hand held measurements of leaf area index

FVC was computed from all methods and compared to LAI of 54 plots at 245 DAS. As shown in Fig. 9, ExG and CIVE had the lowest coefficient of determination with $R^{2} = 0.02$ as on Fig. 9 and 0.02 as in Fig. 9. ACE, *K*-means, ExGR and MFL showed a linear increase with LAI and had the highest coefficient of determination, 0.82, 0.88, 0.91 and 0.92, respectively.

**Fig. 11:**
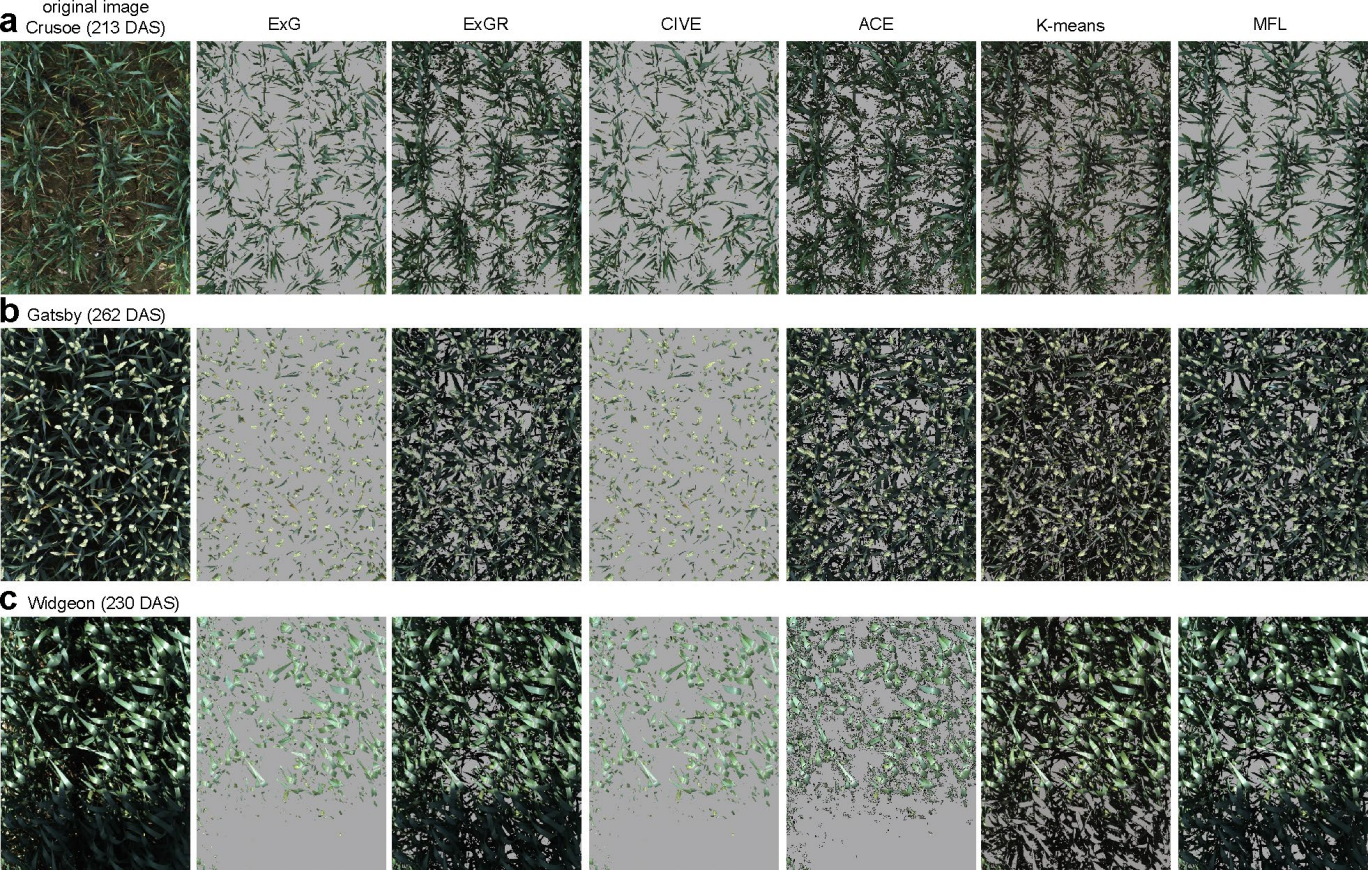
Segmentation results of six methods. The columns from the first to sixth demonstrate the segmentation results by ExG, ExGR, CIVE, *K*-means, ACE, and the proposed MFL method respectively. **a** Crusoe 213 DAS, **b** Gatsby 262 DAS, **c** Widgeon 230 DAS

### Vegetation segmentation of tested methods over a full growing season

Figure 10 presents the fractional vegetation cover of all methods over 33 time points throughout the life-cycle of Crusoe, Gatsby and Widgeon cultivars. ExG and CIVE behaved similarly throughout the season in all three tested varieties with substantial day to day variation. Both tended to underestimate the FVC over the rapid canopy expansion period (197–240 DAS) and over-estimate canopy cover during the senescence period (245–284 DAS). Moreover, examples taken from different timestamps in Fig. 11b, c, clearly illustrated that both methods failed to segment vegetation properly .

ACE showed daily inconsistency over the plant life cycle (Fig. 10). As illustrated in Fig. 11, ACE either generated noise which led to over-segmenting vegetation (Fig. 11a) or failed to segment vegetation adequately, specially when part of the image was in shade (Fig. 11c).

In comparison, ExGR, *K*-means and MFL presented a similar pattern throughout the time series (Fig. 10). From emergence to 185 DAS, ExGR and *K*-means showed more day to day variation compared to MFL for all three tested varieties. From 185 to 284 DAS, ExGR and MFL had similar FVC values in the Gatsby and Maris Widgeon varieties, while *K*-means showed more fluctuation in the same period (Fig. 10b, c). Although the three methods in Fig. 10a, gave similar patterns from 185 to 284 DAS, ExGR and *K*-means had higher values of FVC than MFL. The example taken at 213 DAS (Fig. 11a) showed ExGR and *K*-means segmented background noise as vegetation, which may justify the higher value of FVC.

## Discussion

Analysis of images acquired outdoors is a challenging task, as ambient illumination changes throughout a growing season. Unlike single plants grown in pots within greenhouse facilities, segmenting the vegetation from a field-grown plot is complex due to overlapping leaves, as well as portions of the canopy that are shadowed or displaying high specular reflectance; each of which contribute to an underestimation of vegetation pixels within an image. To be relevant for high-throughput phenotyping in field conditions, vegetation segmentation algorithms must be robust enough to handle dynamic illumination conditions and complex canopy architecture throughout the entire crop life cycle.

### Colour index-based techniques (ExG, ExGR, CIVE)

It has been shown that single colour with automatic thresholding cannot adequately segment vegetation from a complex canopy in dynamic outdoor environments. As presented in all four experiments, although ExG and CIVE are easy to implement and require low computational complexity, they performed poorly, particularly at canopy expansion stage, 197 DAS (Fig. 10). When the contrast between foreground/vegetation and background is high, ExG and CIVE performed similarly to the other methods during the winter period until 185 DAS. As shown in Fig. 10, during canopy expansion when background soil is hardly visible, bimodal pixel distribution dropped (Additional file 1), and the performance of Otsu thresholding deteriorated considerably between 197 and 284 DAS (Figs. 10, 11).

However, ExGR demonstrated a high level of performance unlike results presented elsewhere [3, 12]. It showed a high correlation with LAI and performed consistently over illumination changes during a day (Table 2), as well as in time series (Fig. 10b, c). However, ExGR performed poorly with noisy backgrounds, which led to a high rate of vegetation misclassification (Figs. 6c, 11a) and in high lightness intensity/spectral reflectance.

### Unsupervised learning-based techniques (*K*-means, ACE)

In addition to colour index-based methods, two unsupervised machine learning techniques known as ACE [8] and *K*-means clustering were tested and compared with the proposed model. Although ACE showed a high correlation with LAI, it performed poorly in dynamic outdoor environments in general. In the experiments reported here, ACE computed a low value of $Q_{{ seg}}$ and a high rate of misclassification of vegetation (Fig. 6). ACE also, performed inconsistently in terms of extracting FVC throughout the plant life cycle.

*K*-means achieved good segmentation performance across all conditions with a high rate of $Q_{{ seg}}$ and a low level of misclassification error (Fig. 6). It also performed well in the time series experiment; however, it appeared to over-estimate FVC in certain conditions as illustrated in Crusoe from 197 to 240 DAS due to residual noise (Figs. 10a, 11a). It also showed higher fluctuation in FVC compared to ExGR and MFL during the same periods. Nevertheless, the main drawback of using *K*-means is the iterative process which is computationally expensive. Moreover, the performance of *K*-means clustering depends on the selected number of clusters. The poor choice of cluster numbers may affect the performance of segmenting vegetation considerably.

### Multi-feature supervised learning model (MFL)

Throughout the conditions tested within this study, the MFL method achieved the best performance. It demonstrated the highest quality of segmentation indices with the lowest variation compared to the five other methods and had the lowest misclassification rate. It performed consistently throughout the growth cycle (Fig. 10) under different natural light conditions (Fig. 7) as well as with various backgrounds without pre-defining parameters.

Conversely, the proposed machine learning approach holds the advantage of versatility and could be applied to extract more than just green vegetation, such as yellow/brown organs appearing during senescence, or even for the detection of disease and/or pest symptoms with an adequate training dataset. As already mentioned, the performance of any supervised learning model strongly depends on training datasets. Therefore, in order to have a good model, a substantial set of training data plays an important role. Acquiring a training data is time-consuming and can be subjective. An aim is to expand this study by integrating a semi-adaptive approach to generate bigger and more reliable training datasets semi-automatically; in addition, testing the model on more varieties and different crops is required.

## Conclusion

This study shows that the proposed machine learning approach can be an essential tool for the development of data analysis pipelines in high-throughput field phenotyping. The learning model has shown a great capability to segment vegetation in various environments with various illumination conditions from “simple” to “complex” images. This study also highlighted that the classical colour index-based methods, ExG, CIVE with a single colour thresholding or unsupervised learning models like ACE may not be relevant, when it comes to dynamic illumination conditions.

For the first time, the robustness of vegetation segmentation algorithms (classical and machine learning) were tested along the whole crop life cycle, with increasing canopy complexity within images, as well as under dynamic illumination conditions experienced over multiple seasons. This study highlights that the proposed MFL approach is a relevant tool for time series analysis of field grown crops. The proposed method has a clear advantage over other colour index-based and unsupervised learning approaches, as it can be applied to other types of applications and is not limited to segmenting green vegetation only.

## Additional file

**Additional file 1.**  Vegetation segmentation using ExG + Otsu thresholding The figure shows the histogram of the image and how Otsu set a threshold from the histogram. As shown in the image, when the canopy reaches full maturation, the automatic Otsu thresholding failed to set a threshold properly leads to miss a lot of vegetation.
