# Supplementary figures and images for: Multi-feature machine learning model for automatic segmentation of green fractional vegetation cover for high-throughput field phenotyping
Source: Plant Methods. 2017 Nov 21;13:103. doi: 10.1186/s13007-017-0253-8 (PMC5696775; doi:10.1186/s13007-017-0253-8)

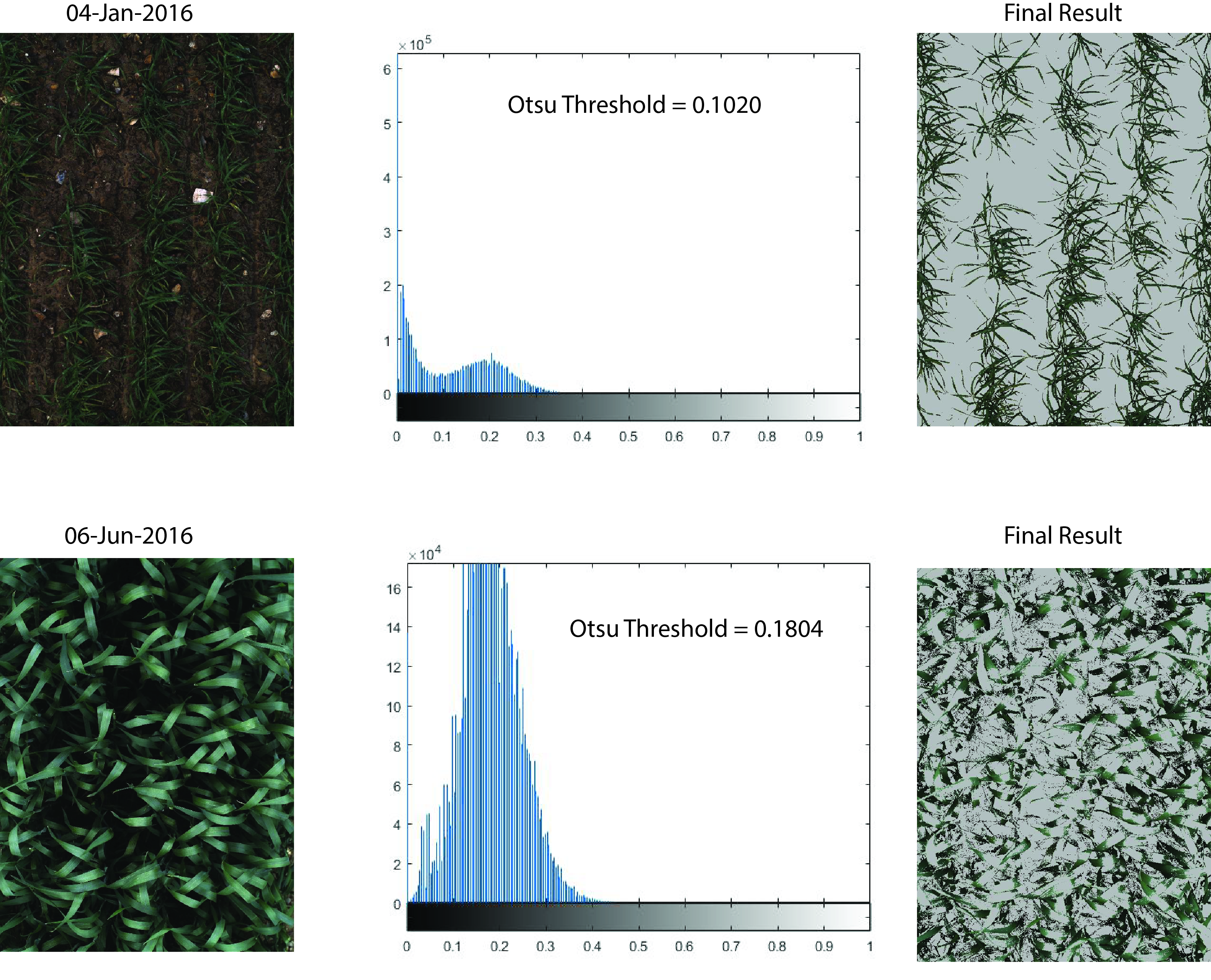

Supplement: Supplementary file 1 — Additional file 1. Vegetation segmentation using ExG + Otsu thresholding The figure shows the histogram of the image and how Otsu set a threshold from the histogram. As shown in the image, when the canopy reaches full maturation, the automatic Otsu thresholding failed to set a threshold properly leads to miss a lot of vegetation. [file 13007_2017_253_MOESM1_ESM.jpg]
